# The level of caveolin-1 expression determines response to TGF-*β* as a tumour suppressor in hepatocellular carcinoma cells

**DOI:** 10.1038/cddis.2017.469

**Published:** 2017-10-12

**Authors:** Joaquim Moreno-Càceres, Daniel Caballero-Díaz, Zeribe Chike Nwosu, Christoph Meyer, Judit López-Luque, Andrea Malfettone, Raquel Lastra, Teresa Serrano, Emilio Ramos, Steven Dooley, Isabel Fabregat

**Affiliations:** 1 Bellvitge Biomedical Research Institute (IDIBELL), Oncobell Program, L‘Hospitalet de Llobregat, Barcelona, Spain; 2 Molecular Hepatology Section, Department of Medicine II, Medical Faculty Mannheim, University of Heidelberg, Mannheim, Germany; 3 Department of Surgery, Liver Transplant Unit, University Hospital of Bellvitge, Barcelona, Spain; 4 Pathological Anatomy Service, University Hospital of Bellvitge, Barcelona, Spain; 5 Department of Physiological Sciences II, University of Barcelona, Barcelona, Spain

## Abstract

Hepatocellular carcinoma (HCC) is a heterogeneous tumour associated with poor prognostic outcome. Caveolin-1 (CAV1), a membrane protein involved in the formation of caveolae, is frequently overexpressed in HCC. Transforming growth factor-beta (TGF-*β*) is a pleiotropic cytokine having a dual role in hepatocarcinogenesis: inducer of apoptosis at early phases, but pro-tumourigenic once cells acquire mechanisms to overcome its suppressor effects. Apoptosis induced by TGF-*β* is mediated by upregulation of the NADPH oxidase *NOX4*, but counteracted by transactivation of the epidermal growth factor receptor (EGFR) pathway. Previous data suggested that CAV1 is required for the anti-apoptotic signals triggered by TGF-*β* in hepatocytes. Whether this mechanism is relevant in hepatocarcinogenesis has not been explored yet. Here we analysed the TGF-*β* response in HCC cell lines that express different levels of CAV1. Accordingly, stable CAV1 knockdown or overexpressing cell lines were generated. We demonstrate that CAV1 is protecting HCC cells from TGF-*β-*induced apoptosis, which attenuates its suppressive effect on clonogenic growth and increases its effects on cell migration. Downregulation of CAV1 in HLE cells promotes TGF-*β*-mediated induction of the pro-apoptotic *BMF*, which correlates with upregulation of *NOX4*, whereas CAV1 overexpression in Huh7 cells shows the opposite effect. CAV1 silenced HLE cells show attenuation in TGF-*β*-induced EGFR transactivation and activation of the PI3K/AKT pathway. On the contrary, Huh7 cells, which do not respond to TGF-*β* activating the EGFR pathway, acquire the capacity to do so when CAV1 is overexpressed. Analyses in samples from HCC patients revealed that tumour tissues presented higher expression levels of *CAV1* compared with surrounding non-tumoural areas. Furthermore, a significant positive correlation among the expression of *CAV1* and *TGFB1* was observed. We conclude that CAV1 has an essential role in switching the response to TGF-*β* from cytostatic to tumourigenic, which could have clinical meaning in patient stratification.

Hepatocellular carcinoma (HCC) is a heterogeneous tumour commonly associated with chronic liver diseases, such as alcoholic and viral hepatitis, and is often preceded by cirrhosis.^1^ Given the lack of an effective therapeutic approach, several studies have focused on molecular targets that can predict either clinical outcome or drug response. Caveolins are a family of membrane proteins required for the formation of membrane invaginations called caveolae. Caveolae are involved in cellular trafficking, and have been proposed as possible sites for mining druggable targets in cancer.^2^ Interestingly, in addition to the role of caveolins in caveolae formation, they also act as scaffolding proteins, and as such modulate intracellular signalling pathways.^3^ Caveolin-1 (CAV1), the mostly studied member of the family (others being CAV2 and CAV3), functions either as a tumour suppressor or as an oncogene, depending on tumour type and cellular context.^3^ Nevertheless, in HCC several evidences propose CAV1 as an important factor determining higher invasive and metastatic phenotypes, as well as poor prognosis.^4, 5, 6^ CAV1 expression has been found to be increased concomitant with HCC progression. This correlates with the fact that overexpression of CAV1 promotes HCC cell growth, increases motility and invasiveness, as well as higher tumourigenic potential *in vivo*.^4, 6^ Interestingly, knockdown of CAV1 in metastatic HCC cells had the opposite effect, suppressing tumour growth and metastatic potential *in vivo*.^6^ Further, CAV1 expression varies with differentiation state of HCC cells: well-differentiated cell lines do not express detectable levels of CAV1, whereas high expression can be found in poorly differentiated HCC cell lines.^7^ However, the mechanism for CAV1 involvement in all these effects is not fully understood yet.

Transforming growth factor-beta (TGF-*β*) is a pleiotropic cytokine that also has a dual role in tumourigenesis. For instance, TGF-*β* acts as a growth inhibitor in early stages of cancer, but promotes progression once cells have acquired the mechanism to overcome its suppressor effect. Thus, in liver tumour cells, TGF-*β* regulates a balance between both pro- and anti-apoptotic signals, which is critical for cell fate decisions.^8^ Cells that circumvent its pro-apoptotic action may undergo epithelial–mesenchymal transition (EMT),^9^ further acquiring increased migratory^10^ and drug resistance capabilities.^11^ Previously, we have shown that mainly poorly differentiated HCC cell lines resist the cytostatic effect of TGF-*β*.^12^ Overall, there is a great interest in a better understanding of the mechanisms that allow liver tumour cells to preferentially respond to TGF-*β* pro-survival signals. CAV1 affects TGF-*β*/-Smad (canonical) and non-Smad (non-canonical) signalling and thus can determine the cellular outcome upon TGF-*β* challenge.^13, 14^ Indeed, CAV1 is required for the non-canonical signalling pathways that mediate anti-apoptotic signals triggered by TGF-*β* in hepatocytes,^15, 16^ although nothing is known about whether it has a similar role in HCC cells.

In this study, we more thoroughly investigated the impact of CAV1 on the TGF-*β* response in HCC cell lines and found out that CAV1 is critical to blunt the tumour-suppressor function of TGF-*β* in HCC cells.

## Results

### CAV1 expression impairs TGF-*β*-induced apoptosis in HCC cell lines

**Figure 1:**
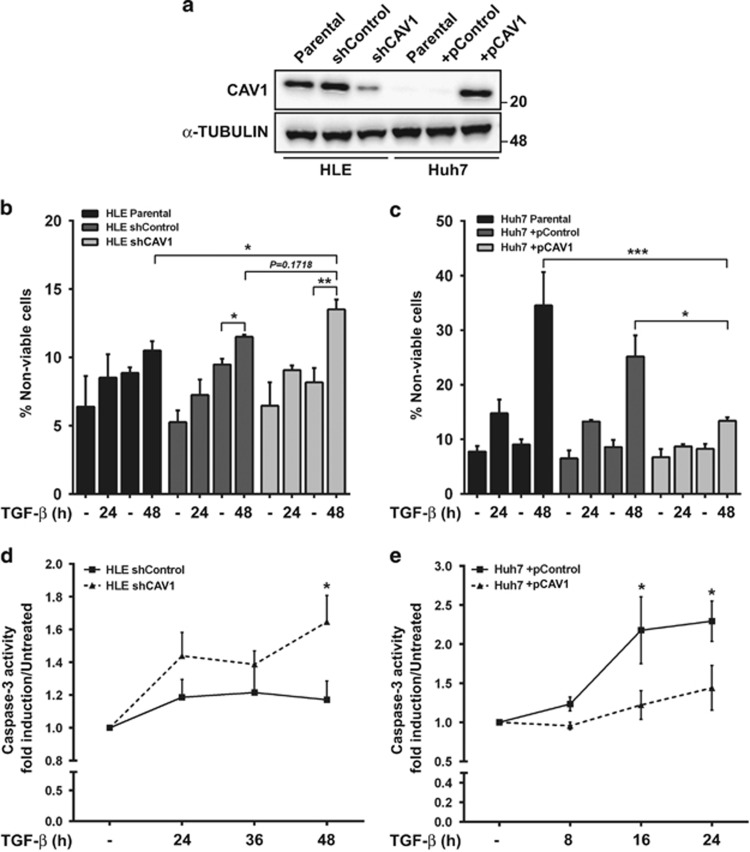
CAV1 expression interferes with TGF-*β*-induced apoptosis in HCC cell lines. HLE parental, HLE shControl, HLE shCAV1, Huh7 parental, Huh7 +pControl and Huh7 +pCAV1 were treated with TGF-*β* (5 ng/ml) at the times shown after previous FBS starvation (2% FBS; 4 h). (**a**) Immunoblot of total protein extracts; *α*-TUBULIN as loading control. A representative experiment is shown. (**b** and **c**) Cell viability measured using Trypan blue staining and expressed as percentage of non-viable cells (*N*=4 for **b** and *N*=3 for **c**). (**d** and **e**) Caspase-3 activity, expressed as fold induction *versus* an untreated control (*N*=6 for **d** and *N*=5 for **e**). Results are expressed as mean±S.E.M. Statistical comparison uses two-way ANOVA with Sidak post-hoc test as shown in the figure: *P<0.05, **P<0.01, ***P<0.001

We first tested whether CAV1 has a role in regulating the balance between pro- and anti-survival signals in HCC cells. On the one hand, we knocked down CAV1 in HLE cells, a poorly differentiated HCC cell line that shows high CAV1 expression (Figure 1a). On the other hand, we overexpressed CAV1 in Huh7 cells, a well-differentiated HCC cell line with low basal CAV1 expression (Figure 1a). In HLE, knockdown of CAV1 increased the percentage of TGF-*β-*induced cell death in comparison with high CAV1-expressing controls, suggesting CAV1 as an antagonist of the TGF-*β*-mediated apoptotic response (Figure 1b). In line with this, overexpression of CAV1 in Huh7 cells significantly suppressed TGF-*β*-induced cell death (Figure 1c). Furthermore, we found that TGF-*β* activation of caspase-3 (a pro-apoptotic mediator) depends on the level of CAV1 expression (Figures 1d and e). These evidences suggest that CAV1 may be protecting HCC cells from TGF-*β* death-inducing signals.

### Overexpression of CAV1 enables clonogenic growth and promote cell migration under TGF-*β* stimulation

**Figure 2:**
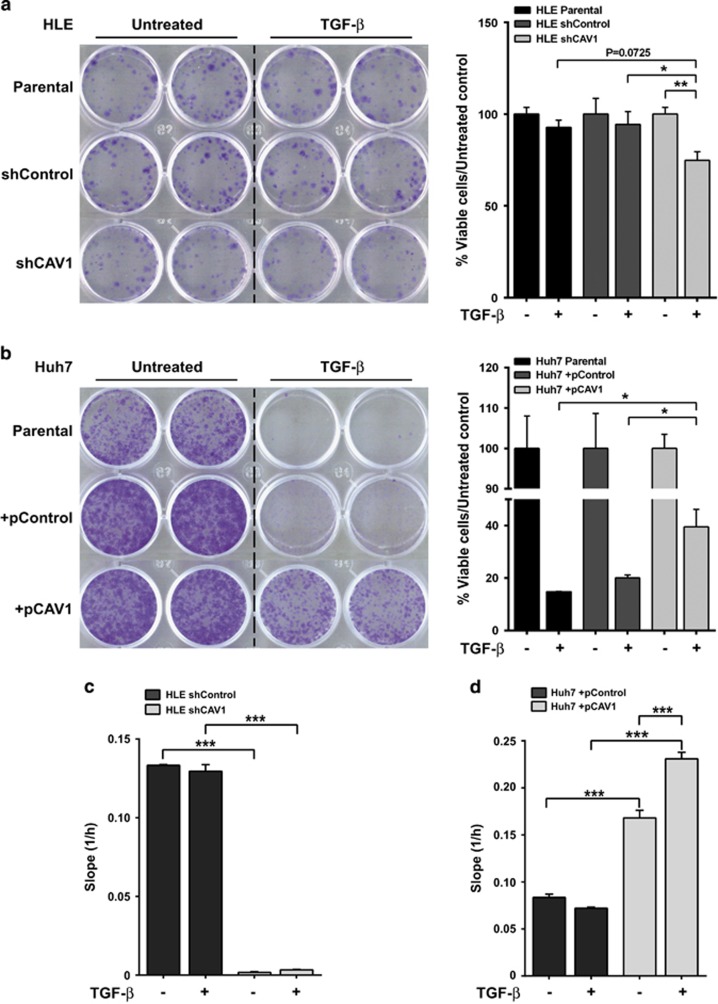
CAV1 expression levels in HCC cell lines determine the clonogenic ability in presence of TGF-*β* and alter their migratory capacity. (**a** and **b**) HLE parental, HLE shControl, HLE shCAV1, Huh7 parental, Huh7 +pControl and Huh7 +pCAV1 were treated with TGF-*β* (5 ng/ml) for 1 week in complete medium (10% FBS). Crystal violet stained colonies indicate clonogenic growth; a representative experiment is shown (left), and quantification is presented from three independent experiments (right). (**c** and **d**) Cell migration in real-time was analysed by the xCELLigence RTCA. Cells were treated with TGF-*β* (2 ng/ml) for 72 h. Migration rate was determined by analysing the slope of the lineal interval, between 10 and 35 h in HLE cells and between 15 and 35 h in Huh7 cells (*N*=6 from two independent experiments). Results are expressed as mean±S.E.M. Statistical comparison uses two-way ANOVA with Sidak post-hoc test as shown in the figure: *P<0.05, **P<0.01, ***P<0.001

We next evaluated if CAV1 expression interferes with anti-proliferative action and facilitates tumourigenic activity of TGF-*β*. First, we measured clonogenic growth upon TGF-*β* stimulation in HCC cell lines with modulation in CAV1 expression. As expected from our previous study,^12^ TGF-*β* had no effect on clonal proliferation of HLE cells, whereas knockdown of CAV1 decreased clonogenic growth in presence of TGF-*β* (Figure 2a). Consistently, the inhibitory effect of TGF-*β* on clonal growth of Huh7 was counteracted in the state of ectopic CAV1 expression (Figure 2b). Cell cycle arrest is among the cytostatic effects induced by TGF-*β*.^17^ Hence, we next performed flow cytometry analyses to determine whether CAV1 would influence TGF-*β* effects on cell cycle. HLE cells did not respond to TGF-*β* inhibiting cell cycle progression (Table 1a; Supplementary Figure 1A). In contrast, Huh7 exhibited the characteristic features of cell cycle arrest: an increase in the percentage of cells in G_0_/G_1_ phase and a decrease in S and G_2_/M phases. However, this was uninfluenced by ectopic CAV1 expression (Table 1b; Supplementary Figure 1B). Finally, as one of the main tumourigenic actions of TGF-*β* is inducing cell migration, we explored whether silencing or overexpressing *CAV1* alters the TGF-*β*-migratory capability in these cells. Results indicated that silencing CAV1 *per se* is enough to decrease the high migratory capability of HLE cells (Figure 2c). Furthermore, *CAV1* overexpression promotes basal migration of Huh7 cells and, interestingly, sensitised cells to the pro-migratory effects of TGF-*β* (Figure 2d).

Taken together, results indicate that CAV1 expression attenuates the suppressive effect of TGF-*β* on clonogenic growth, which is explained by its effects on impairing apoptosis, as it does not influence the effects of TGF-*β* on cell cycle arrest. In addition, expression of CAV1, probably through its effects mediating apoptosis resistance, favours the HCC cell response to the pro-migratory effects of TGF-*β*.

### CAV1 modulates TGF-*β*-dependent regulation of *BMF*, *BIM* and *NOX4*

**Figure 3:**
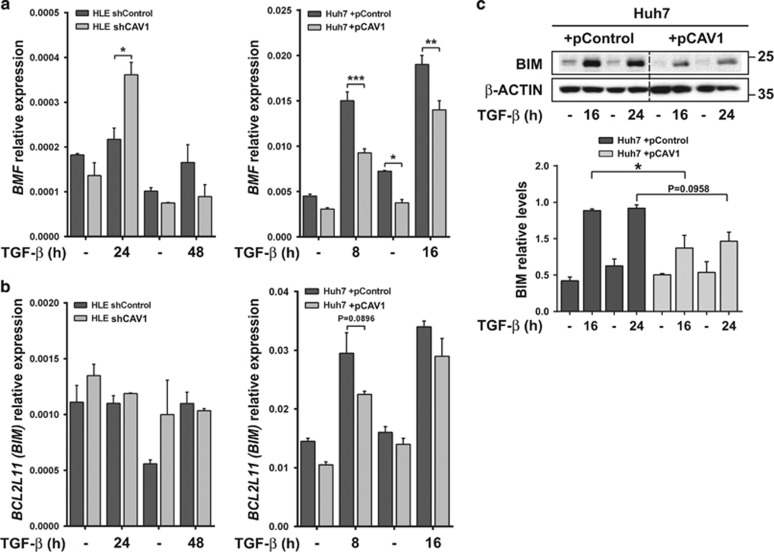
Expression of BCL-2 family genes is altered by CAV1 in HCC. HLE shControl, HLE shCAV1, Huh7 +pControl and Huh7 +pCAV1 were treated with TGF-*β* (2 ng/ml) at the times shown after previous FBS starvation (2% FBS; 4 h). (**a** and **b**) Relative expression levels of *BMF* and *BIM*; *L32* was used as house-keeping gene (*N*=2). Results are expressed as mean±S.E.M. (**c**) Upper panel: immunoblot of total protein extracts; *β*-ACTIN is presented as loading control. A representative experiment is shown (*N*=3). Lower panel: densitometric analysis of BIM relative levels; results are mean±S.E.M. of three independent experiments and are expressed as absolute values. Statistical comparison was done by two-way ANOVA with Sidak post-hoc test as shown in the figure: *P<0.05, **P<0.01, ***P<0.001

TGF-*β* controls expression of different apoptosis regulatory proteins at both transcriptional and post-transcriptional levels. For instance, TGF-*β* in context of an apoptotic response upregulates the pro-apoptotic BCL-2 family members, BIM (*BCL2L11*) and BCL-2-modifying factor (*BMF*).^8, 18^ Hence, we wondered whether CAV1 has modulatory impact on TGF-*β* mediated regulation of *BIM* and *BMF*. In HLE with intrinsically high basal CAV1, TGF-*β*-mediated *BMF* induction is disrupted (Figure 3a, left panel). However, the downstream TGF-*β* pathway branch could be reactivated in HLE upon CAV1 knockdown, resulting in significantly induced *BMF* expression (Figure 3a, left panel). Consistently, TGF-*β*-mediated induction of *BMF* was decreased in Huh7 cells with ectopic CAV1 overexpression (Figure 3a, right panel). For *BIM*, no differences were observed in response to TGF-*β* in HLE cells at mRNA (Figure 3b, left panel) and protein level (data not shown), regardless of presence or absence of CAV1 (Figure 3b, left panel). In Huh7, CAV1 overexpression prevented upregulation of *BIM* (*BCL2L11*) at mRNA and protein levels after short term TGF-*β* treatment (Figure 3b, right panel and Figure 3c). These results suggest *BMF* as one TGF-*β*-regulated BCL-2 family member consistently affected by CAV1 in the two HCC cell lines.

**Figure 4:**
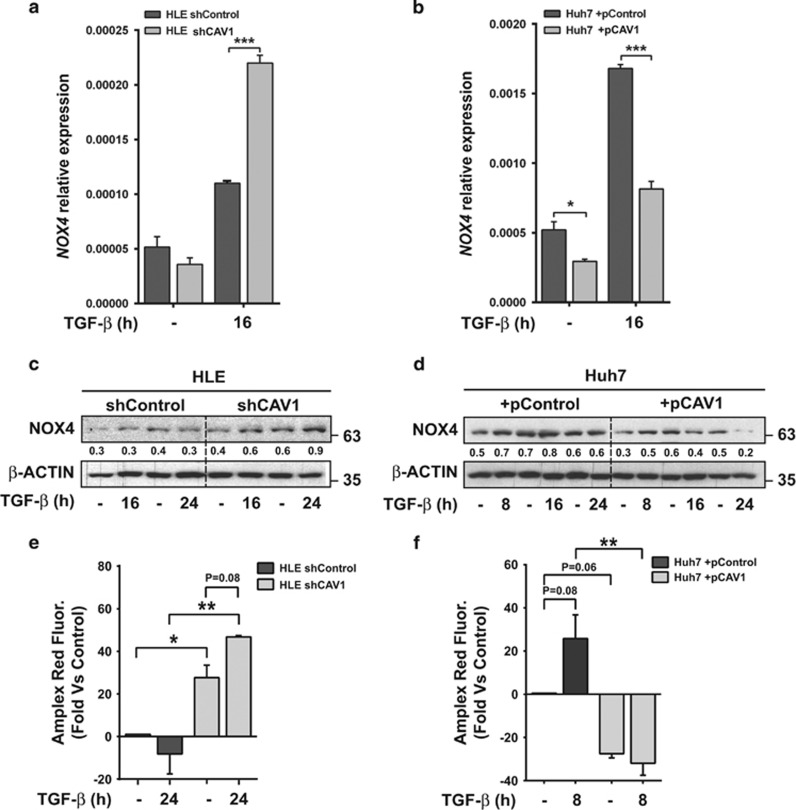
*NOX4* upregulation after TGF-*β* treatment in HCC cell lines is decreased by CAV1. HLE shControl, HLE shCAV1, Huh7 +pControl and Huh7 +pCAV1 were treated with TGF-*β* (2 ng/ml) at the times shown after previous FBS starvation (2% FBS; 4 h). (**a** and **b**) Relative expression levels of *NOX4*; *L32* was used as house-keeping gene (*N*=3). Results are expressed as mean±S.E.M. (**c** and **d**) Immunoblot of total protein extracts; *β*-ACTIN was used as loading control. A representative experiment is shown, whose quantification (densitometry of the specific band relative to the *β*-ACTIN levels) is incorporated below the band. (**e** and **f**) Amplex Ultra Red Fluorescence as a measure of ROS production, expressed as relative percentage *versus* untreated HLE shControl cells or Huh7 pControl cells (*N*=6 from two independent experiments). Statistical comparison was done using two-way ANOVA with Sidak post-hoc test as shown in the figure: *P<0.05, **P<0.01, ***P<0.001

Transcriptional regulation of *BMF* via TGF-*β* requires expression of the NADPH oxidase NOX4,^18^ a known factor in cellular oxidative stress that also mediates pro-apoptotic effects in liver cells.^19^ We found that *NOX4* levels are very low in HLE cells, and are only slightly induced by TGF-*β*. However, CAV1 knockdown resulted in a significant increase in TGF-*β* stimulated *NOX4* mRNA levels (Figure 4a). In the case of Huh7 cells, interestingly, *NOX4* expression was significantly decreased in CAV1-overexpressing cells under basal conditions (Figure 4b). Furthermore, TGF-*β*-mediated increase in *NOX4* mRNA levels was strongly impaired by CAV1 overexpression (Figure 4b). Increased expression of *NOX4* upon TGF-*β* stimulation in CAV1 knockdown HLE cells was also observed at the protein level (Figure 4c) and correlated with enhanced reactive oxygen species (ROS) levels under basal conditions and in response to TGF-*β* (Figure 4e). In Huh7, overexpression of CAV1 induced a decrease in NOX4 protein levels, including upon TGF-*β* stimulation (Figure 4d), which correlated with a decrease in ROS levels and lack of response to TGF-*β* in terms of ROS production (Figure 4f). Worthy to mention that silencing CAV1 in HLE cells increased basal NOX4 levels (in the absence of TGF-*β*), correlating with higher ROS, and overexpressing CAV1 in Huh7 decreased NOX4 levels both at the mRNA and protein level, correlating with decreased ROS. Thus, basal NOX4 expression is also influenced by the CAV1 levels.

Together, these data suggest that high expression of CAV1 in HCC cells impairs the induction of *NOX4* expression by TGF-*β* and the consequent ROS-dependent upregulation of pro-apoptotic genes, in particular *BMF*.

### CAV1 is required for TGF-*β*-induced epidermal growth factor receptor (EGFR)-mediated survival signals in HCC cell lines

**Figure 5:**
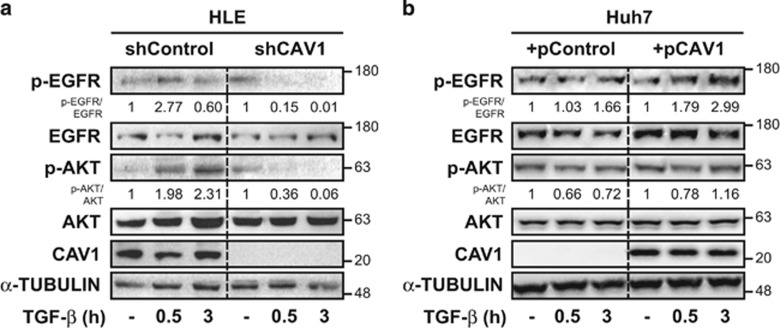
CAV1 facilitates EGFR/AKT survival axis in HCC cells. HLE shControl, HLE shCAV1, Huh7 +pControl and Huh7 +pCAV1 were treated with TGF-*β* (2 ng/ml in **a** or 5 ng/ml in **b**) or HB-EGF (20 ng/ml) at the times shown after previous FBS starvation (2% FBS; 4 h). (**a** and **b**) Immunoblot of total protein extracts; *α*-TUBULIN was used as loading control. A representative experiment is shown

Previous studies had revealed that in hepatocytes and liver tumour cells, upregulation of *NOX4* by TGF-*β* depends on both canonical Smad-dependent, as well as other non-canonical pathways. Indeed, *NOX4* is upregulated by TGF-*β* through a Smad-dependent mechanism, but transactivation of the EGFR by TGF-*β* impairs its effects on *NOX4* expression in a PI3K-dependent mechanism.^20^ For this reason, we evaluated the role of CAV1 on TGF-*β*-induced EGFR activation in HCC cells. In HLE cells, we observed subtle EGFR activation after TGF-*β* treatment, which further resulted in activation of the PI3K pathway (Figure 5a). This effect was inhibited in CAV1-suppressed HLE cells (Figure 5a), indicating that CAV1 expression is required for EGFR transactivation by TGF-*β*. In Huh7 cells, no EGFR transactivation is observed upon TGF-*β* stimulation (Figure 5b), in line with this cell line’s high sensitivity to TGF-*β*-mediated cell death. CAV1 overexpression sensitised Huh7 cells to TGF-*β*/EGFR phosphorylation pathway branch (Figure 5b). Furthermore, in HLE, TGF-*β*-induced AKT survival signalling was blunted upon CAV1 depletion, whereas TGF-*β*-mediated inhibition of AKT phosphorylation in Huh7 was abrogated upon CAV1 overexpression. These results suggest that CAV1 is required for TGF-*β* activation of non-canonical pathways that mediate anti-apoptotic signals.

Worthy no note that silencing *CAV1* decreased cell proliferation in HLE cells in presence of FBS or the EGFR ligand heparin binding EGF-like growth factor (HB-EGF) (Supplementary Figure 2A) and *CAV1*-overexpressing Huh7 cells showed a higher proliferation rate in comparison with control cells (Supplementary Figure 2B). Therefore, these data suggest that CAV1 confers to the HCC cells advantages in terms of proliferation, probably facilitating response to growth factors in terms of proliferation and survival.

### *CAV1* is overexpressed in tumoural tissues from HCC patients and its expression correlates with *TGFB1* levels

**Figure 6:**
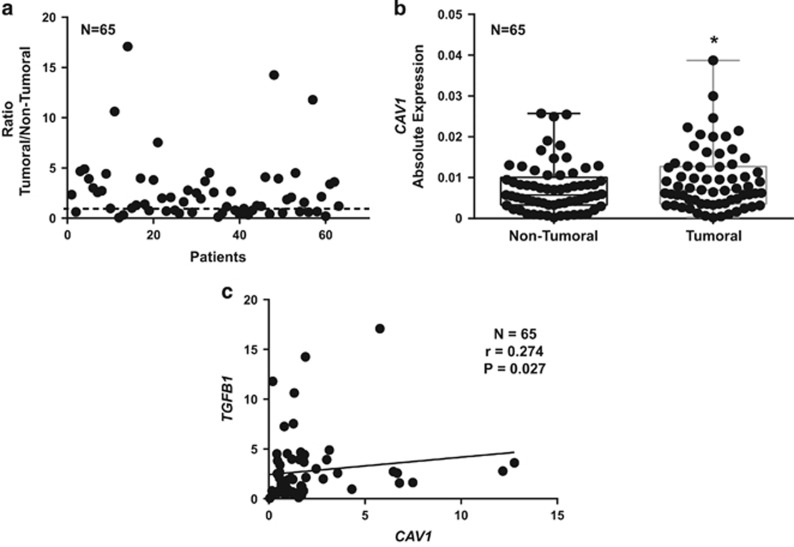
Analysis of *CAV1* and *TGFB1* expression in HCC tumoural and non-tumoural tissues. qRT-PCR analysis of mRNA levels of *CAV1* in non-tumoural and tumoural tissue. (**a**) Detail of the relative expression of each one of the HCC tumour tissues analysed *versus* its respective surrounding tissue (*n*=65). Black line represents cut-off (relative expression=1). (**b**) Each dot represents relative expression of each HCC tumour tissue *versus* its respective surrounding tissue. Statistical comparison was done using Wilcoxon matched-pairs signed rank test: *P<0.05. (**c**) Spearman correlation analysis among *TGFB1* and *CAV1* expression analysed by qRT-PCR in the cohort of 65 samples from HCC patients

We finally decided to examine the potential translational relevance of the previous results in HCC. We analysed the expression of *CAV1* and *TGFB1* in a cohort of 65 HCC patients, in tumoural and surrounding areas. Tumoural tissue presented higher expression levels of *CAV1* compared with surrounding non-tumoural tissue (Figure 6a and b). Moreover, we found a significant positive correlation among the changes in the expression of *CAV1* and *TGFB1* (Figure 6c). From public data (Mas Liver Dataset from Oncomine: Supplementary Figure 3), we found that expression of *TGFB1* and *CAV1* increased from the cirrhotic status and correlated with virus hepatitis C (VHC) infection. These data indicate that co-expression of *CAV1* and *TGFB1* frequently occurs in HCC.

**Figure 7:**
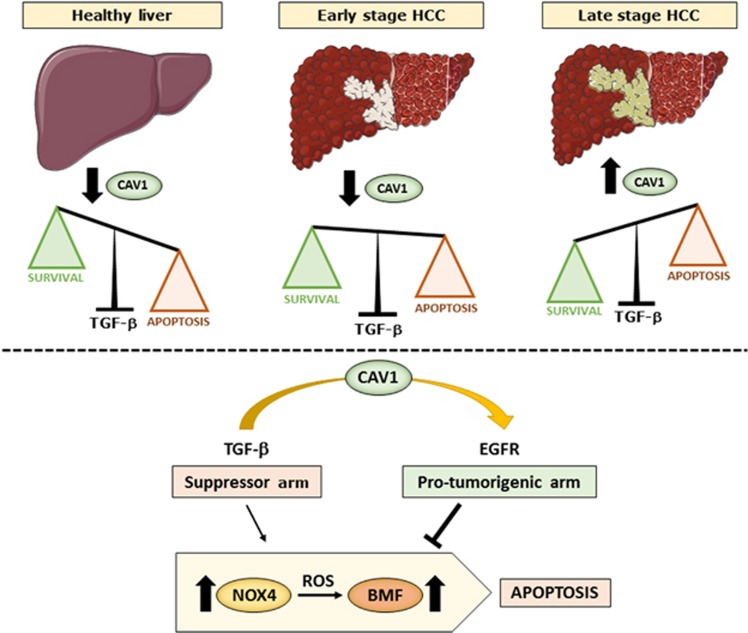
CAV1 orchestrates the TGF-*β* response in HCC. Whereas in healthy liver, CAV1 levels are very low, its expression increases through the hepatocarcinogenic process, being very high in poorly differentiated liver tumour cells (i.e., HLE). In those late stage liver tumour cells, high expression of CAV1 inhibits TGF-*β*-induced apoptosis through its interaction with the EGFR pathway, which impairs *NOX4* upregulation by TGF-*β* and BMF-dependent apoptosis

Altogether, our results indicate that CAV1 expression increases during HCC progression, coinciding with enhanced *TGFB1* levels. Under these circumstances, CAV1 could mediate survival in liver tumour cells through its interaction with the EGFR pathway, which impairs *NOX4* upregulation by TGF-*β* and *BMF*-dependent apoptosis (Figure 7).

## Discussion

TGF-*β* signalling participates in all stages of liver disease progression, from initial damage via inflammation and fibrosis to cirrhosis and cancer. During liver tumourigenesis, TGF-*β* may behave as a suppressor factor at early stages; however, strong evidences suggest that overactivation of its signalling could later contribute to tumour progression, once cells escape from its cytostatic effects.^21^ For these reasons, targeting the TGF-*β* signalling pathway is being explored to counteract liver disease progression.^22^

Similarly, different evidences indicate that CAV1 may also act both as tumour-suppressor or pro-tumourigenic protein.^23^ Mechanisms underlying such pleiotropic, and sometimes antagonistic, effects of CAV1 function is a research line of great interest.^24, 25^ Anti-apoptotic functions of CAV1 have been reported in different experimental models.^3^ Related to liver tumour cells, CAV1 may block apoptosis under stress conditions, that is, in serum-deprived media, in the human hepatoblastoma HepG2 cells.^26^ CAV1 also confers resistance to anoikis in hepatoma cells by activating the insulin-like growth factor-1 pathway.^27^ However, little is known about how CAV1 may regulate TGF-*β*-induced apoptosis in liver cells.

Here, we show that in a well-differentiated HCC cell line that does not express CAV1 and which is sensitive to the pro-apoptotic effect of TGF-*β*, ectopic overexpression of CAV1 counteracts suppressive TGF-*β* stimuli. On the contrary, in a poorly differentiated HCC cell line with high CAV1 expression, knockdown of this protein increases susceptibility to TGF-*β*-mediated cell death. Importantly, CAV1 expression does not have any effect on the suppressor response to TGF-*β* on cell cycle arrest. Resistant HLE cells are not sensitised for TGF-*β-*mediated inhibition of cell proliferation upon CAV1 knockdown. Further, ectopic CAV1 expression, which significantly counteracts TGF-*β* anti-apoptotic signals in Huh7, has no obvious impact on TGF-*β*-mediated cell cycle arrest. However, the remarkable results obtained on clonogenic growth prove that CAV1 mediated resistance to TGF-*β*-induced cell death in HCC cells has marked consequences with regard to loss of response to the suppressor effects of this cytokine. These results indicate that apoptosis is the main anti-tumourigenic effect of TGF-*β* in HCC cells. Furthermore, CAV1 is required to allow cells to respond to this cytokine inducing cell migration. Indeed, CAV1 switches the function of TGF-*β* from suppressor to pro-tumourigenic.

We also show that in HLE cells, downregulation of CAV1 promotes TGF-*β*-mediated induction of pro-apoptotic *BMF*, whereas on the contrary, in Huh7 cells overexpression of CAV1 reduces TGF-*β-*induced *BMF* expression. We also observe regulation of *BIM*, particularly at the protein levels, indicating that its regulation is at least partly post-transcriptional. These results correlate with previous findings, showing that CAV1 regulates expression of BCL-2 family proteins in hepatocytes.^15^ TGF-*β* exerts its pro-apoptotic effects in hepatocytes also through induction of *NOX4*, which produces ROS and oxidative stress.^20, 28^ An influence of NOX4 on regulation of members of the BCL-2 family was previously suggested.^8, 18^ Here we propose that basal levels of NOX4 in HCC cells are influenced by CAV1 levels. More interestingly for our study, induction of *NOX4* by TGF-*β* depends on CAV1 expression levels. Indeed, overexpression of CAV1 substantially blocks *NOX4* induction, whereas CAV1 knockdown shows the opposite effect. Hence, mechanisms that counteract TGF-*β*-induced *NOX4* expression are facilitated by upregulated CAV1.

The EGFR pathway is a crucial mechanism, by which TGF-*β* mediates cell survival in hepatocytes,^29^ and previous results indicate that CAV1 is required for this connection.^16, 30^ EGF counteracts *NOX4* upregulation by TGF-*β* in hepatocytes,^20^ whereas, in contrast, different evidences indicate that EGFR inhibition induces expression of *NOX4.*^31,32^ Here we show that CAV1 silenced HLE cells show attenuation in TGF-*β*-induced EGFR transactivation and activation of the PI3K/AKT pathway. On the contrary, Huh7 cells, which do not respond to TGF-*β* activating the EGFR pathway, acquire the capacity to do so, when CAV1 is overexpressed. These results indicate that the important role proposed for CAV1 in mediating the EGFR non-canonical signalling pathway of TGF-*β* in hepatocytes^15, 16^ is also present in liver tumour cells. As a consequence, our data suggest that the level of CAV1 expression in HCC patients may be an important determinant for defining the tumour cell response to TGF-*β* signalling. In this sense, here we show evidences for a positive correlation between the changes in the expression of *CAV1* and *TGFB1* in a cohort of HCC patients. Expression of *TGFB1* and *CAV1* increases from the cirrhotic status and correlated with VHC infection. These data indicate that co-expression of *CAV1* and *TGFB1* frequently occurs in HCC, as was previously suggested in other kind of tumours, such as prostate cancer.^33^ This would have marked consequences on the HCC cell response to TGF-*β*. As previously mentioned, CAV1 overexpression is associated with HCC tumourigenesis and metastasis.^4, 5, 6^ Here we proposed for the first time that CAV1 could mediate its action in HCC, at least in part, through modulating tumour cell response to TGF-*β*.

In conclusion, our results suggest that high expression of CAV1 in liver tumour cells triggers switching the response of TGF-*β* from a suppressor to a tumourigenic factor. As further knowledge about the mechanisms that favour the pro-tumourigenic actions of TGF-*β* is requested for rationalising TGF-*β* directed drugs to treat HCC in human, we suggest expression of CAV1 as a potential molecular marker for better stratification of patients prone for such therapy.

## Materials and methods

### Reagents and antibodies

Human recombinant TGF-*β*1 was from Calbiochem (La Jolla, CA, USA) or from Peprotech (Hamburg, Germany). Foetal bovine serum (FBS) was from Sera Laboratories International (Cinder Hill, UK). Anti-NOX4 rabbit polyclonal antiserum was raised by Sigma–Genosys (Suffolk, UK) against a peptide corresponding to the C-terminal loop region (amino acids 499–511). Specificity was tested by ELISA with the purified peptide.^18^ This NOX4 antibody is currently available from Merck Millipore (Billerica, MA, USA; cat. no. ABC459). The other antibodies used were: mouse anti-*β*-ACTIN (clone AC-15) from Sigma-Aldrich (St. Louis, MO, USA); rabbit anti-phospho-AKT (Ser473) (D9E) XP, rabbit anti-AKT, rabbit anti-phospho-EGFR (Tyr1068) (D7A5) XP and rabbit anti-EGFR were from Cell Signaling Technology (Beverly, MA, USA); mouse anti-CAV1 and rabbit anti-BIM were from BD Pharmingen (Franklin Lakes, NJ, USA); mouse anti-*α*-TUBULIN (4G1I) was from AbCam (Cambridge, UK). Secondary antibodies: ECL mouse IgG, and rabbit IgG, HRP-linked antibodies from GE Healthcare (Buckinghamshire, UK).

### Ethics statement in the work with human HCC tissues

Human tissues were collected with the required approvals from the Institutional Review Board (Comité Ético de Investigación Clínica, Hospital Universitario de Bellvitge) and patient's written consent conformed to the ethical guidelines of the 1975 Declaration of Helsinki. A total of 65 patients were included in the study and both tumour and non-tumour tissues were collected.

### Cell culture conditions

The HCC cell lines HLE and Huh7 were obtained from the Japanese Collection of Research Bioresources (JCRB) Cell Bank (Osaka, Japan). For cell culture, cells were grown in Dulbecco’s modified Eagle’s medium from Lonza (Basel, Switzerland), supplemented with 10% FBS, penicillin (120 *μ*g/ml), streptomycin (100 *μ*g/ml) and amfoterycin B (2.5 *μ*g/ml) and maintained in a humidified atmosphere of 37ºC, 5% CO_2_. For experiments, cells at 60% confluence were serum starved (2% FBS) for 4 h and treated with TGF-*β*1 (2 ng/ml – Calbiochem or 5 ng/ml – PeproTech).

### Stable knockdown and overexpression of CAV1

Lentiviral constructs of GFP+ *CAV1* overexpression and knockdown (*shCAV1)* with their respective non-targeting controls, were kind gifts from Dr. Carsten Herskind (Universitätsklinikum, Mannheim, Germany). To generate stable cells, we transduced Huh7 (low *CAV1* expressing) with *CAV1* overexpression vector, whereas knockdown was achieved in HLE, which constitutively express high level of *CAV1*. For transduction, sub-confluent cells were seeded in complete growth media (12-well multiwell plate) and cultured overnight. The next day, the culture media were aspirated off and replaced with a fresh one containing polybrene to a final concentration of 8 *μ*g/ml. Lentiviral particles were then added to the respective wells at a multiplicity of infection (MOI) of 10. After overnight culture, we remove the media, replaced with normal culture media and further incubated the cells for another 24 h before expansion. Positive cells were selected by fluorescent activated cell sorting (FACS) using BD FACSAria I (BD Bioscience, Heidelberg, Germany).

### Analysis of cell viability

Cell viability was analysed using Trypan blue. Cells were trypsinised while collecting the media. After 5- min centrifugation at 1200 r.p.m., cells were resuspended in 50 *μ*l PBS + 1:10 v/v Trypan blue. Viable and non-viable (blue dyed) cells were counted in a Neubauer chamber, counting eight squares for condition, with duplicates. Results are expressed as percentage of non-viable cells.

### Crystal violet assay

Cells were stained with crystal violet solution (0.2% (w/v) in 2% ethanol). Stained cells were lysed by adding SDS 10%. By spectrophotometric analysis, the absorbance was measured at 595 nm. Results were expressed as percentage of viable cells *versus* untreated control in each condition or time zero.

### Analysis of caspase-3 activity

Fluorimetric analysis of caspase-3 activity was determined using the caspase-3 fluorogenic substrate AC-DEVD-AFC from Biomol (Hamburg, Germany). Briefly, after TGF-*β* treatment, media were collected and dead cells on it were centrifuged at 2000 r.p.m. for 5 min at 4 °C. Then cells on the plate were washed with HBSS and scrapped in 100 *μ*l of lysis buffer (50 mM HEPES, 100 mM NaCl, 0.1% CHAPS, 1 mM DTT, 100 *μ*M EDTA, pH 7.4). Cells from both media and plate were assembled in the same tube. Afterward 20 *μ*l of protein lysate were mixed with 70 *μ*l of reaction buffer (50 mM HEPES, 100 mM NaCl, 0.1% CHAPS, 10 mM DTT, 100 *μ*M EDTA, 10% glycerol, pH 7.4) and finally 10 *μ*l of substrate were added (previously diluted 1 : 20 in reaction buffer) and plated into a 96-well plate that was incubated at 37 °C for 60 and 120 min, at these times fluorescence was measured at excitation 400 nm and emission 505 nm. Protein concentration was determined using a Bio-Rad commercial kit (Munich, Germany). Results were calculated as fluorescence units per *μ*g of protein and then expressed as fold induction *versus* untreated control.

### Clonogenic assay

Low number of cells (500 for HLE and 1000 for Huh7 cells per well) were plated in 12-well multiwell plates (22.1 mm diameter wells). TGF-*β* was added in complete media (10% FBS), and it was maintained during 1–2 weeks (changing media every third day). After the time of culture required the cell media were removed, cells were washed twice with HBSS and the remaining viable adherent cells were stained with crystal violet solution.

### DNA content/cell cycle analysis

After incubation with TGF-*β*, cell media were recovered, so detached cells were taken, and the remaining cells on the plate were washed twice with PBS. Then, cells were detached using Trypsin-EDTA (0.05%) and added to the recovered media for centrifugation (5 min at 1500 r.p.m.). The pellet was resuspended in 300 *μ*l of PBS 1X and was added drop by drop into 70 *μ*l of cold (stored at –20 ºC) 100% ethanol, in constant motion (gently applied with a vortex), in order to fix cells. At this point, samples were stored at –20 °C. Then, samples were centrifuged at 1500 r.p.m. during 5 min at 4°C. The pellet was washed twice in HBSS and finally air-dried and resuspended in 250 *μ*l PBS containing 10 *μ*g/ml of RNAse. After incubation during 20 min at 37 °C, propidium iodide (PI) was added at a final concentration of 0.01 mg/ml. Following this process, PI incorporation was directly proportional to the amount of DNA contained by each cell. Finally, this suspension was analysed in a flow cytometer BD FACS Canto II from Becton-Dickinson (BD Bioscience). Cell cycle analysis was carried out using the software BD FACSDIVA from BD Biosciences (Franklin Lakes, NJ, USA). DNA content: 2C: G_0_/G_1_ phases; 4C: G_2_/M phases; >2C and <4C: S phase.

### RNA isolation, cDNA synthesis and qRT-PCR

E.Z.N.A. Total RNA Kit II (Omega Bio-Tek, Norcross, GA, USA) was used for total RNA isolation from both, cell lines and human tissues. Reverse transcription (RT) was carried out using the High Capacity Reverse Transcriptase kit (Applied Biosystems, Foster City, CA, USA), with 500 ng of total RNA from each sample for complementary DNA synthesis. For Real-Time quantitative PCR, expression levels were determined in duplicate in a LightCycler 480 System, using LightCycler 480 SYBR Green I Master Mix (Roche, Basel, Switzerland). Reactions were performed with the following human-specific primers:


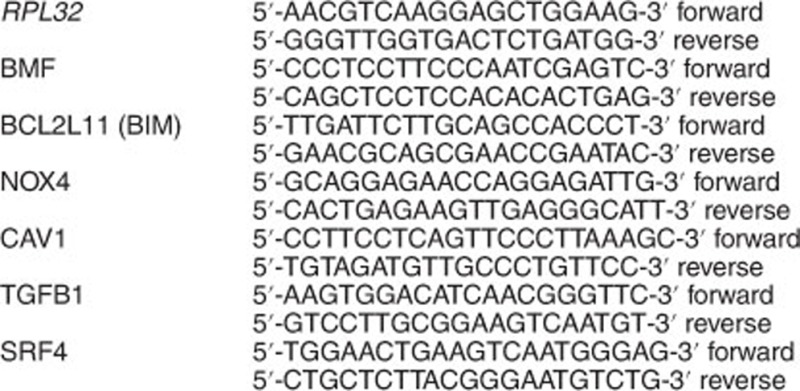


### Immunoblot analysis

Total protein extracts and western blot procedure were carried out as previously described.^34, 35^ Primary antibodies were used at 1 : 1000, except *β*-actin (1 : 5000). Secondary antibodies were used at 1 : 5000. Protein concentration was measured using a Bio-Rad commercial kit. Densitometric analysis of protein bands intensity was performed using ImageJ software (NIH, Bethesda, MD, USA).

### Reactive oxygen species analysis

Extracellular H_2_O_2_ was measured in intact cells using horseradish peroxidase-linked Amplex Ultra Red (Invitrogen, Carlsbad, CA, USA). Briefly, Amplex Ultra Red (50 *μ*M for HLE cells and 100 *μ*M for Huh7 cells) and horseradish peroxidase (0.1 U/ml for HLE cells and 0.2 U/ml Huh7 cells) were added to the cellular samples for 2 h. Fluorescence readings were made at the times indicated in the corresponding figure, in duplicate in a 96-well plate at ex/em 530/590 nm using 100 *μ*l samples of medium. Fluorescence was measured in a Fluostar Optima microplate fluorescence reader (from BMG LABTECH, Ortenberg, Germany) and expressed as percentage of control after correction for cell number (crystal violet assay) with Amplex Ultra Red.

### Real-time migration assay

xCELLigence System was used for real-time monitoring of cell migration. CIM plates (ref. 05 665 817 001, ACEA Biosciences, Inc., San Diego, CA, USA) were placed onto the Real-Time Cell Analyzer (RTCA) station (xCELLigence System, Roche, Mannheim, Germany) for monitoring cell migration. First, both sides of the membrane were coated with collagen type IV solution (25.5 *μ*g/cm^2^) (ref. C7521, Sigma-Aldrich). In all, 100*μ*l of a suspension containing 4x10^5^ cells/ml with 2% FBS were seeded onto the top chamber of a CIM plate and treated with TGF-*β*. The lower chamber contained medium with 10% FBS as a chemoattractant. Cell migration was continuously monitored throughout the experiments by measuring changes in the electrical impedance at the electrode/cell interface, as a population of cells migrated from the top to the bottom chamber. Continuous values every 15 min were represented as cell index (CI), a dimensionless parameter, which reflects a relative change in measured electrical impedance, and quantified as a slope (h^-1^) of the first hours.

### Statistics

All data represent at least three experiments and are expressed as the mean ± S.E.M. Differences between groups were compared using Student’s *t*-test (when comparing two groups), one-way ANOVA with Tukey’s multiple comparison test (when comparing more than two groups and considering one independent variable), or two-way ANOVA with Sidak post-hoc test (when comparing differences between groups considering two independent variables). For patient samples, statistical comparison was done using Wilcoxon matched pairs signed rank test and the Spearman correlation analysis. All statistical tests were conducted using GraphPad Prism (GraphPad, San Diego, CA, USA). Differences were considered statistically significant at *P<0.05, **P<0.01 and ***P<0.001.

## Figures and Tables

**Table 1:** Caveolin-1 expression levels in HCC cell lines do not impact TGF-*β*-induced cell cycle arrest

	**Parental**		**shControl**		**shCAV1**	
	**Untreat. 48h**	**TGF-*****β*** **48h**	**Untreat. 48h**	**TGF-*****β*** **48h**	**Untreat. 48h**	**TGF-*****β*** **48h**
(a) *HLE*						
G0/G1	65.15±2.14	58.86±1.27	47.86±2.16	45.13±1.56	65.27±3.90	60.01±1.76
S	13.29±1.33	18.14±0.69	6.96±0.42	8.96±0.73	13.66±0.65	18.87±1.01
G2/M	19.27±0.63	20.43±0.70	41.76±2.23	41.33±2.59	16.68±0.85	18.80±0.59

	**Parental**		**+pControl**		**+pCAV1**	
	**Untreat. 48h**	**TGF-*****β*** **48h**	**Untreat. 48h**	**TGF-*****β*** **48h**	**Untreat. 48h**	**TGF-*****β*** **48h**
(b) *HUH7*						
G0/G1	65.88±3.48	76.25±0.04	57.43±2.39	68.42±2.02*	59.59±1.20	74.43±2.97*
S	15.75±3.66	8.35±1.49	15.08±2.98	6.23±1.27	21.94±1.52	9.30±1.37**
G2/M	16.70±0.34	8.65±1.09	24.73±1.69	16.77±0.78*	16.89±0.43	12.76±0.72*

HLE parental, HLE shControl, HLE shCAV1, Huh7 parental, Huh7 +pControl and Huh7 +pCAV1 were treated with TGF-*β* (5 ng/ml) for 48 h after previous FBS starvation (2% FBS; 4 h). (a and b) Quantification of cell cycle phases (G_0_/G_1_, S and G_2_/M), expressed as mean±S.E.M. (*N*=3). Statistical comparison using Student’s *t*-test to compare control *versus* TGF-*β* treatment in each cell line: *P<0.05, **P<0.01
