# Supplementary material for: The level of caveolin-1 expression determines response to TGF-β as a tumour suppressor in hepatocellular carcinoma cells
Source: Cell Death Dis. 2017 Oct 12;8(10):e3098–. doi: 10.1038/cddis.2017.469 (PMC5680590; doi:10.1038/cddis.2017.469)
Supplement: Supplementary Figures [file cddis2017469x1.pdf]

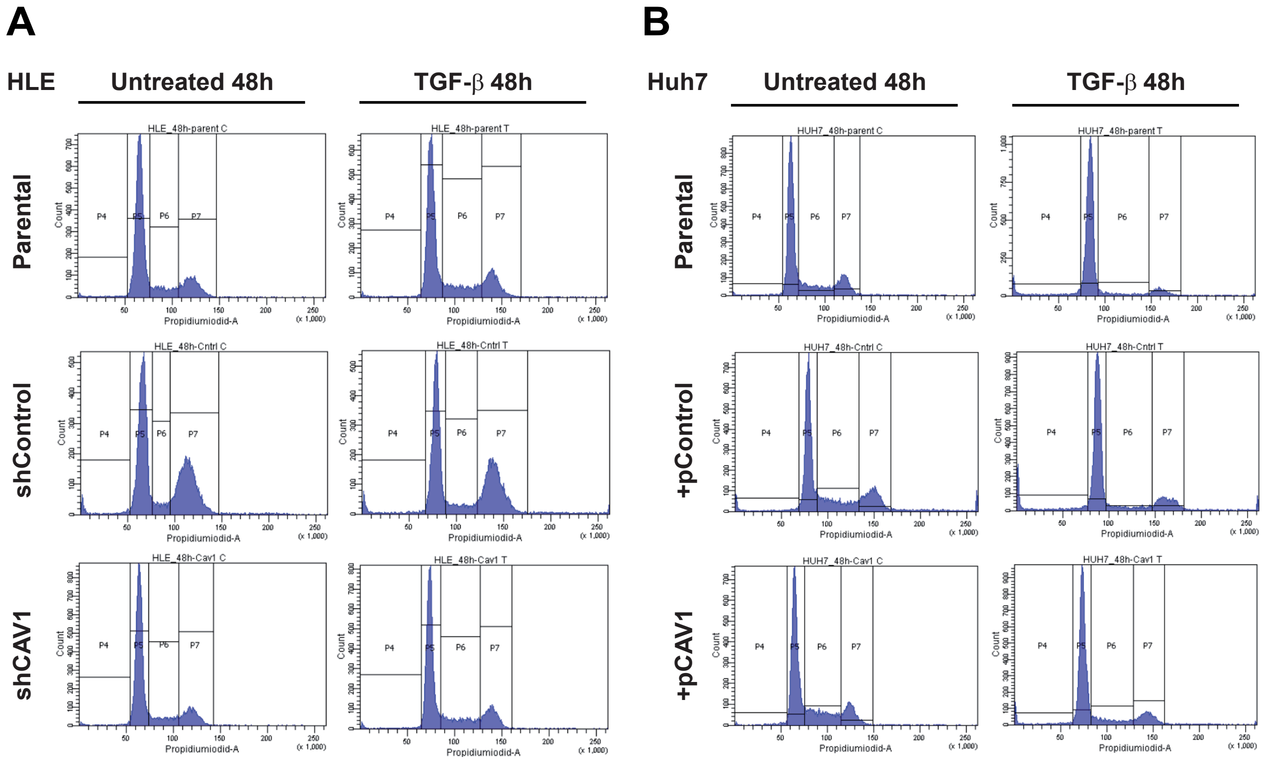

Supplementary figure 1. Caveolin-1 expression levels in HCC cell lines do not impact TGF-β-induced cell cycle arrest (cytometry histograms). HLE parental, HLE shControl, HLE shCAV1, Huh7 parental, Huh7 +pControl and Huh7 +pCAV1 were treated with TGF-β (5 ng/mL) for 48 h after previous FBS starvation (2% FBS; 4 h). A and B: Cytometry histograms depicting the different phases of cell cycle (G_0/G_1 is P5, S is P6 and G_2/M is P7). A representative experiment is shown (N=3).

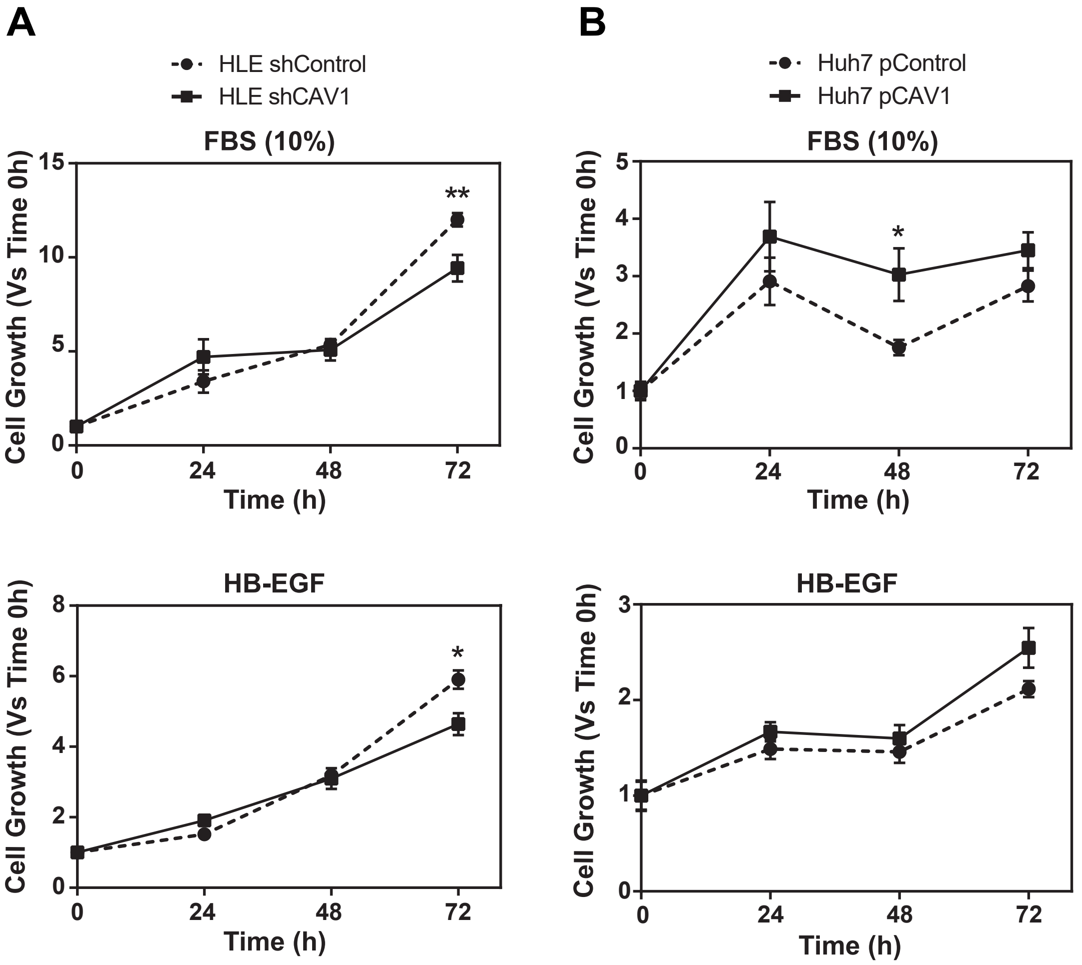

Supplementary figure 2. Caveolin-1 expression levels in HCC cell lines alters proliferation ability. A: HLE shControl, HLE shCAV1 were treated with TGF-β (2 ng/mL) or HB-EGF (20 ng/mL) at the times shown after previous FBS starvation (2% FBS; 4 h) B: Huh7 +pControl and Huh7 +pCAV1 were treated with TGF-β (2 ng/mL) or HB-EGF (20 ng/mL) at the times shown after previous FBS starvation (2% FBS; 4 h). Results are expressed as mean of 3 independent experiments ± S.E.M. Statistical comparison was done using two-way ANOVA with Sidak post-hoc test as shown in the figure: * $p<0.05$, ** $p<0.01$.

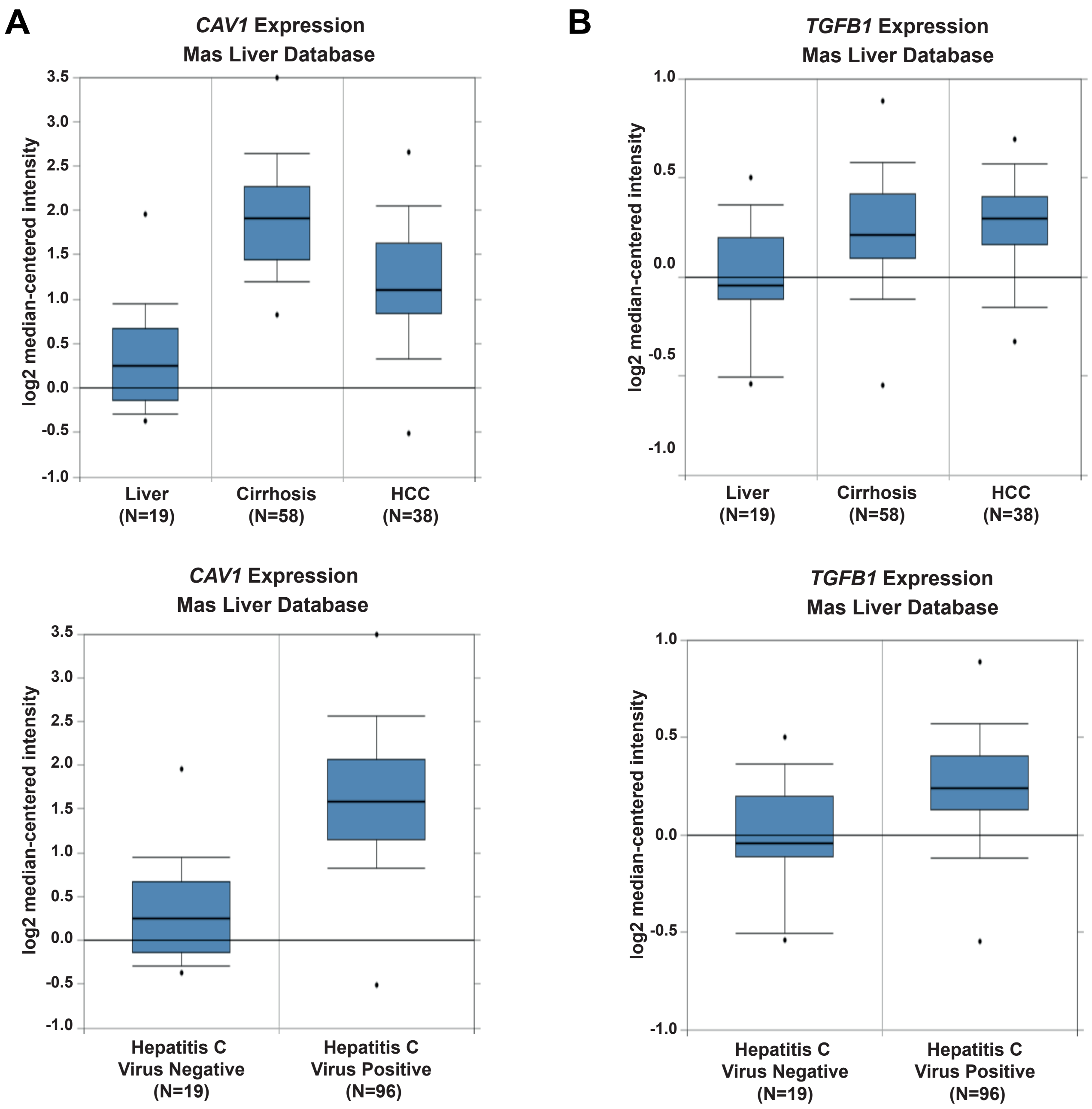

Supplementary figure 3. Expression of *TGFB1* and *CAV1* increases from the cirrhotic status and correlates with VHC infection. A: *CAV1* mRNA expression B: *TGFB1* mRNA expression. Data were collected from Mas Liver Dataset from Oncomine (N=115). Reference Database Oncomine: Mas VR, Maluf DG, Archer KJ, Yanek K, Kong X, Kulik L, Freise CE, Olthoff KM, Ghobrial RM, McIver P, Fisher R. Genes involved in viral carcinogenesis and tumor initiation in Hepatitis C Virus-induced Hepatocellular Carcinoma. (2009) Mol Med. Mar-Apr;15(3-4):85-94. doi: 10.2119/molmed.2008.00110.
